# Editorial: The Role of Neuroinflammation in Chronic Pain Development and Maintenance

**DOI:** 10.3389/fphar.2021.821534

**Published:** 2021-12-23

**Authors:** Francesco De Logu, Serena Boccella, Francesca Guida

**Affiliations:** ^1^ Department of Health Sciences, Clinical Pharmacology and Oncology Section, University of Florence, Florence, Italy; ^2^ University of Campania Luigi Vanvitelli, Napoli, Italy

**Keywords:** pain, inflammation, chronic pain, nervous system, inflammatory cell

Inflammation is originated by tissue injury and triggers several biochemical reactions that sensitize the nervous system for pain perception. Chronic inflammation induces adaptive changes that can cause altered pain signal processes. Indeed, inflammatory mediators provokes structural and functional changes in the peripheral or central sensory circuits, resulting in pain like behaviors, such as hyperalgesia, allodynia, and spontaneous pain. Accumulating evidence suggests that central sensitization is driven by neuroinflammatory processes mediated by activation/proliferation of resident microglia and glial cells with the consequent release of inflammatory mediators; furthermore, several recent studies are relating the role of non-neuronal cells of the peripheral nervous system with the modulation of neuroinflammation involved in the development and maintenance of chronic pain.

Currently treatments for chronic pain are quite unsatisfying. Therefore, there is a need for research aimed at discovering novel biological targets for new pharmacological approaches. Recently the authors collectively participated in the study of neuropathic pain and the role of HCAR2 expressed by Schwann cells in a sciatic nerve ligation model. (Boccella et al.).

In this Research Topic, different signaling implicated in neuroinflammation and chronic pain and possible therapeutic approaches have been described:

➢ Borbèly et al. investigate the role of tachykinin hemokinin-1 (HK-1) in arthritis by using different mouse models mimicking pathophysiology of rheumatoid arthritis in humans and integrative methodology (functional, *in vivo* optical imaging, cell cultures) with special emphasis on pain. They provide the first evidence that HK-1 mediates several arthritic inflammatory mechanisms and pain by direct activation of primary sensory neurons not via its classical NK1 receptor.
➢ Li et al., summarize the current knowledge of the role of microglia during sepsis by showing the contribute of these cells in long-term sensorial and cognitive deficits. The Authors appropriately illustrate the activation of microglia in the Central nervous system by describing specific neurotransmissions and molecular pathways which may promote the cognitive damage and chronic pain during sepsis.
➢ Pacini et al. test the effects of the natural antioxidant thioctic acid which is clinically used as racemic mixture. The Authors report preclinical and clinical evidences suggesting positive properties of thioctic acid in the treatment of low back pain with a more relevant efficacy of (+)-thioctic acid as compared to (+/−)-thioctic acid on pain, on time of onset of therapeutic effects.
➢ De Caro et al., show the anti-inflammatory and analgesic properties of Perampanel, the selective non-competitive AMPA receptor antagonist in acute (tail flick and hot plate tests) and chronic pain (Chronic constriction injury). They also suggest the involvement of the cannabinergic system (i.e., activation of cannabinoid type 1 receptor, CBR1) in Perampanel actions.
➢ Scuteri et al., provide a systematic review and meta-analysis assessing the efficacy of essential oils in pain. They describe the literature search conducted on databases relevant for medical scientific literature, i.e., PubMed/MEDLINE, Scopus, and Web of Science following the PRISMA (Preferred Reporting Items for Systematic reviews and Meta-Analyses) criteria. Among a total number of 30 studies included, three investigated neuropathic pain conditions.
➢ Borges et al., review recent clinical and preclinical studies investigating the epidermal growth factor receptor (EGFR) signaling pathway in chronic pain states. EGFR inhibitors are known for their use as cancer therapeutics however recent evidence suggested their clinical use for treating chronic pain. Here, the Authors provide an overview of EGFR signaling, highlighting possible mechanisms by which EGFR and its ligands may influence pain hypersensitivity and modulate inflammatory mediators of pain.
➢ Brik et al., investigate the behavioral and biochemical changes after intra-articular injection of sodium monoiodoacetate (MIA). This model mimics the degenerative changes observed in osteoarthritic patients. The Authors have characterized grading changes after injection of 1, 2 or 3 mg of MIA over a 28-day period. They report significant dose- and time-dependent changes in both gene expression and protein secretion levels of inflammatory factors.
➢ Mazzitelli et al., provide a direct evidence for the modulation of pain-like behaviors and spinal neuronal activity by basolateral amygdala-central nucleus (BLA–CeA) signaling and central nucleus-corticotropin releasing factor (CeA–CRF) neurons on under normal conditions and in an arthritis pain model.
➢ Ma et al., reveal the (fat-mass and obesity-associated protein) FTO-triggered epigenetic mechanism of matrix metallopeptidase 24 (MMP24) upregulation in the spinal cord after spinal nerve ligation (SNL). They show that blocking the SNL-induced increase of MMP24 in the spinal cord mitigate pain hypersensitivity both in the development and maintenance phase without altering the basal/acute responses or locomotor functions. The Authors suggest that MMP24 may be an endogenous initiator of neuropathic pain and could be a potential target for this disorder’s prevention and treatment.
➢ Bruno et al., indicate that β-adrenergic receptors (β-ARs), mainly β2-and β3-ARs, contribute to tumor proliferation and progression and cancer-associated pain in a mouse model of cancer pain generated by the para-tibial injection of K7M2 osteosarcoma cells. This study highlights the ability of β-ARs antagonists in modulating both tumor growth and the neuroinflammation, showing a new therapeutic targets for cancer therapy and associated pain.

